# Allicin, the Odor of Freshly Crushed Garlic: A Review of Recent Progress in Understanding Allicin’s Effects on Cells §

**DOI:** 10.3390/molecules26061505

**Published:** 2021-03-10

**Authors:** Jan Borlinghaus, Jana Foerster (née Reiter), Ulrike Kappler, Haike Antelmann, Ulrike Noll, Martin C. H. Gruhlke, Alan J. Slusarenko

**Affiliations:** 1 Department of Plant Physiology, RWTH Aachen University, 52056 Aachen, Germany; jana.reiter@rwth-aachen.de (J.F.); noll@bio3.rwth-aachen.de (U.N.); Martin.Gruhlke@rwth-aachen.de (M.C.H.G.); 2 School of Chemistry and Molecular Biosciences, University of Queensland, St. Lucia, QLD 4072, Australia; u.kappler1@uq.edu.au; 3 Institute of Biology-Microbiology, Freie Universität Berlin, 14195 Berlin, Germany; haike.antelmann@fu-berlin.de

**Keywords:** TRPA1, Yap1, Keap1-Nrf2, DNA gyrase, glutathione, glutathione reductase, lung pathogens, *Haemophilus influenzae*, apoptosis, cancer, ornithine decarboxylase, *Arabidopsis*, yeast

## Abstract

The volatile organic sulfur compound allicin (diallyl thiosulfinate) is produced as a defense substance when garlic (*Allium sativum*) tissues are damaged, for example by the activities of pathogens or pests. Allicin gives crushed garlic its characteristic odor, is membrane permeable and readily taken up by exposed cells. It is a reactive thiol-trapping sulfur compound that *S*-thioallylates accessible cysteine residues in proteins and low molecular weight thiols including the cellular redox buffer glutathione (GSH) in eukaryotes and Gram-negative bacteria, as well as bacillithiol (BSH) in Gram-positive firmicutes. Allicin shows dose-dependent antimicrobial activity. At higher doses in eukaryotes allicin can induce apoptosis or necrosis, whereas lower, biocompatible amounts can modulate the activity of redox-sensitive proteins and affect cellular signaling. This review summarizes our current knowledge of how bacterial and eukaryotic cells are specifically affected by, and respond to, allicin.

## 1. Introduction

**Scheme 1:**
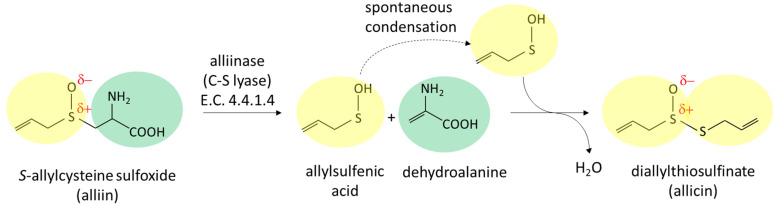
Enzyme-catalyzed biosynthesis of allicin. The internal dipole within the molecule is shown in red.

Garlic (*Allium sativum*) is an internationally appreciated culinary ingredient in many dishes and an economically important agricultural crop in several countries. It is well known that when an intact garlic clove is chopped or crushed it instantly releases a typical pungent bouquet or odor. The characteristic odor of garlic is due to volatile organic sulfur-containing compounds (VOSCs) that are rapidly produced upon tissue damage and diffuse into the air. The major contributor to the odor of freshly crushed garlic is allicin (diallyl thiosulfinate, preferred IUPAC name = *S*-prop-2-en-1-yl prop-2-ene-1-sulfinothioate). Garlic releases VOSCs to defend itself against attacks by pathogens and pests and its antibacterial principle was identified as allicin by Cavallito in 1944 [1,2]. In vivo allicin forms after cellular damage when the enzyme alliinase, which in intact cells is localized in the vacuole, mixes with the substrate alliin which is located in the cytosol (Scheme 1) [3,4,5]. Alliin (*S*-allylcysteine sulfoxide) is an odorless non-protein amino acid. It has been reported that alliinase is predominantly located in bundle sheath cells in bulbs, leaves, and inflorescence axes in the garlic plant [6]. Other alkyl-cysteine sulfoxides are also present in the cytoplasm of garlic cells and can serve as alliinase substrates, yielding amongst others, dimethyl, dipropyl and mixed alkyl thiosulfinates. Nevertheless, allicin accounts for 60–80% of the total thiosulfinates formed by damaged garlic [3]. Alliinase (*S*-alkyl-l-cysteine *S*-oxide alkyl-sulfenate-lyase, E.C. 4.4.1.4) readily converts its substrates to the corresponding sulfenic acids, which condense spontaneously to produce thiosulfinates (Scheme 1) [7]. The reactions are very rapid and are 97% complete within 30 s at 23 °C. Furthermore, a single clove of garlic (approximately 10 g) can produce up to 5 mg of allicin, illustrating the large evolutionary investment the plant places in this defense molecule [8]. An easy and efficient in vitro synthesis protocol for >98% pure allicin and approximately 90% yield has been published [9].

The odor of crushed garlic is usually described as ‘pungent’, which the shorter Oxford English dictionary defines as “biting, caustic or piquant, and affects the skin and the organs of taste and smell with a prickling sensation” [10]. VOSCs are known for their intense and unique olfactory properties, in many cases being detectable by some animal species in ppb. Even the relatively insensitive human nose can detect VOSCs in the low ppm range [7,11].

Garlic-derived preparations were shown to have insect-repellent effects and to discourage the feeding of some birds, for example European starlings. The garlic preparations tested against starlings contained mixed diallyl, dimethyl and allyl-methyl polysulfanes but no allicin [12]. Nevertheless, allicin has repellent effects on herbivores and has been shown to stimulate a subset of TRPA1 (transient receptor potential) non-selective ion channels in pain-sensing (nociceptive) neurons [13,14]. The TRPA1 ion channel, also known as the ‘wasabi receptor’, is activated by a wide range of electrophiles including allyl isothiocyanate, the active ingredient of wasabi [15]. Allyl isothiocyanate activates TRPA1 by covalent modification of a cysteine residue within the cytoplasmic N terminus in TRPA1 [16]. Due to its thiol-reactivity (see later), it seems likely that allicin acts similarly when it stimulates TRPA1-bearing neurons. Furthermore, it was shown to be the same particular subset of TRPA1 neurons that were stimulated by both allyl isothiocyanate and allicin [13]. Activation of TRPA1 results in local inflammation and pain and can evoke innate fear behavior in the sensory host, and it is no surprise therefore that allicin acts as an effective deterrent of herbivory [11,17]. In addition, plants can influence one another and other organisms by using volatile signals which act as allelochemicals, i.e., secondary products that affect the growth, survival or reproduction of other organisms [18]. In this sense, it could be argued that allicin, which has been shown experimentally to inhibit root growth of *Arabidopsis* seedlings (see Section 2.4.1), might also be considered to be an allelochemical [19,20].

Allicin is a colorless liquid at room temperature, but is sometimes described as pale yellow likely due to contamination with trace amounts of elemental sulfur. Because of its relatively high *M*_r_ (162) and the internal dipole within the molecule, allicin would not be expected to have a high vapor pressure. Nevertheless, despite its predicted low volatility allicin is a very odoriferous molecule and small quantities entering the gas phase are easily detected by smell. This becomes obvious every time garlic is chopped or crushed in the kitchen. Unfortunately, the actual vapor pressure of allicin, like its boiling point, is so far not measurable experimentally because upon heating allicin decomposes into a plethora of other VOSCs. For example, heating allicin or garlic, e.g., during cooking, leads to the production of polysulfanes and other physiologically active molecules like ajoene (see Section 4) [21,22]. The dynamic chemistry of the low molecular weight sulfur compounds in biological extracts makes their accurate quantitative and qualitative analysis difficult, and the effects of heating are particularly important. This is often overlooked and is a frequently met flaw in reports purporting to analyze the composition of *Allium* extracts or vapors by gas chromatography (GC) rather than, for example, by liquid chromatography coupled with mass spectrometry (LC-MS). The unavoidable heating step associated with GC inevitably degrades allicin and leads to false composition profiles not reflecting the natural situation.

An important descriptor for a pharmacologically active molecule is its log*P* or *K*_ow_ value. This is an indicator of how a molecule partitions between the non-polar solvent octanol and the polar solvent water. A log*P* value of 1 reflects a 10-fold higher concentration in octanol than water and a value of 0.1 reflects the opposite. Allicin’s lipophilic nature is indicated by its calculated log*P* of 1.35 and it is readily membrane permeable and easily taken up by cells [23]. Similar to the structurally related dimethyl sulfoxide (DMSO) molecule, allicin has been shown to create transient pores as it crosses membranes. Temporary pore formation leads to a transient depolarization, i.e., a decrease in membrane potential (∆*E*_m_) [24]. This effect was observed both in artificial lipid bilayers, where proteins and other potentially allicin-reactive molecules are absent, as well as in biological membranes. Therefore, membrane permeabilization appears to be a physical effect, like that shown for DMSO [25], and is unrelated to any chemical reactivity of allicin [24].

**Scheme 2:**
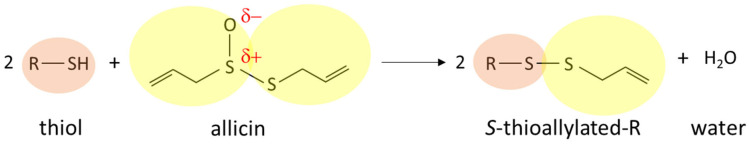
Reaction of allicin with thiols. Two mol of reduced glutathione, other low molecular weight thiol, or accessible cysteine thiols in proteins (R—SH), can react with one mol of allicin to yield two mol of *S*-thioallylated product.

Allicin is a reactive sulfur species (RSS) and a potent thiol-trapping reagent, rapidly reacting with glutathione (GSH) to yield *S*-allylmercaptoglutathione (GSSA). Thus, allicin depletes the cellular GSH pool and reacts with accessible cysteine thiols in proteins by *S*-thioallylation (Scheme 2) [21,26,27,28,29,30,31]. This reaction is key to allicin’s biological activity. Reversible oxidation and reduction of protein-thiols is central to the regulation of many processes in cells [32].

Effects of allicin on living cells can range from its being a dose-dependent biocide to it subtly modifying important cell-signaling pathways and cellular functions at lower, biocompatible doses. The thiol-disulfide exchange reaction (TDER) of a thiol with a disulfide is well described and is important in cellular protein chemistry and signaling [33]. The *S*-thioallylation of a protein thiol by allicin (Scheme 2) can be viewed as a form of TDER. Both the divalent sulfur atom and the sulfinyl sulfur atom are electrophilic but most of the chemistry occurs by nucleophilic attack on the divalent sulfur, with the sulfinyl sulfur being the leaving group when the *O*-atom is protonated. The polarized bond between the *O*- and *S*-atoms of the sulfinyl group significantly weakens the disulfide bond in allicin and makes it more reactive than a simple disulfide towards target nucleophilic thiol groups. Unlike in a standard TDER, the electrons end up in water. Nevertheless, the newly formed disulfide bond can be reduced back to a thiol just like any other protein disulfide, for example by thioredoxins or glutaredoxins, or potentially even by glutathione reductase. In support of this notion, we have recently shown that *S*-allylmercaptoglutathione, produced when allicin reacts with GSH, is a substrate for glutathione reductase [34]. Because allicin is such an effective thiol-trapping reagent it is interesting to know which proteins are *S*-thioallylated in cells by allicin. This is important not only in relation to allicin’s well documented antimicrobial effects [35], but also because the consumption of uncooked garlic, as well as breathing in garlic fumes, exposes cells in the body to allicin. Identifying which proteins become *S*-thioallylated and to what degree, is potentially of great significance in light of the manifold claims of the health benefits of consuming garlic (reviewed in [21]), and its increasing use in plant protection [36].

The purpose of this review is to summarize recent progress in our knowledge of the cellular effects of allicin in bacteria and eukaryotes and builds on our earlier 2014 review [19].

## 2. Allicin and Oxidative Stress in Cells and Organisms

Chemically, allicin clearly reacts as an oxidant when it *S*-thioallylates -SH groups. However, allicin is often described as an antioxidant, particularly in the diet. There are two aspects of allicin’s properties and mode of action that need to be considered in relation to this apparent contradiction. Firstly, from the chemical point of view, an antioxidant describes a substance that prevents or slows down the oxidation of another molecule, and in this regard, it has been shown that allicin readily undergoes a Cope elimination reaction at room temperature to form 2-propenesulfenic acid, a very potent antioxidant [37]. Interestingly, 2-propenesulfenic acid (allylsulfenic acid) is thus both a precursor (Scheme 1) and a decomposition product of allicin [37]. Secondly, from a nutritional and physiological point of view, cells respond to mild oxidative stress by activating oxidative stress protection responses, which prime the cells to be more resistant to subsequent, greater oxidative insults [38,39,40,41,42]. The detection of an oxidative threat to cells usually occurs via specialized sensor proteins, like Keap-1 in mammals, containing oxidation-sensitive cysteine residues [43]. Oxidation of these sensor cysteines leads to a cascade of phase II oxidative stress protective cellular responses. Detailed examples of how allicin interacts with the oxidative-stress-sensing machinery in some model organisms are given in the next section.

### 2.1. The Yeast Yap1 Oxidative Stress Sensor System

**Figure 1:**
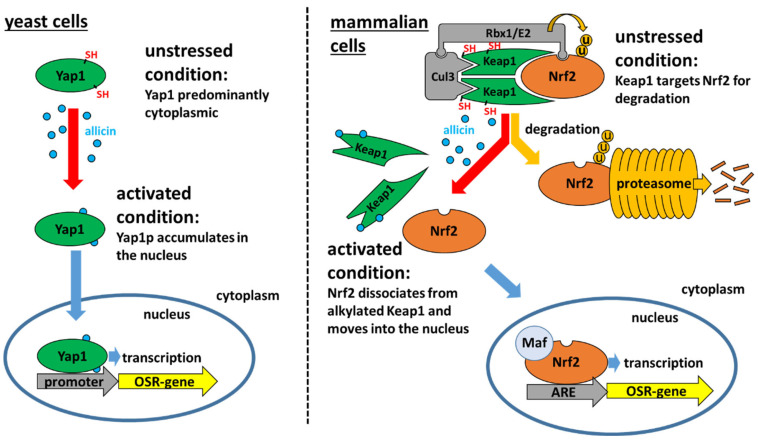
Sensing oxidative stress by specific thiol oxidation in yeast and mammalian cells. The sensing of low *M*_r_ electrophilic oxidants that cause oxidative stress is predominantly based on the oxidation of sensitive cysteine thiols, e.g., in Yap1 in yeast and in Keap1-Nrf2 in mammals. Allicin stimulates the expression of OSR genes in both yeast and mammals by a similar mechanism.

The oxidative stress response (OSR) in yeast (*Saccharomyces cerevisiae*), a model fungus, is centrally regulated by the Yap1 transcription factor which senses H_2_O_2_ in a Gpx3-dependent mechanism via the oxidation of redox-sensitive cysteines [44]. Yap1 regulates the expression of genes important for antioxidant metabolism, e.g., genes on the GSH biosynthetic pathway, glutathione reductase, glutaredoxins, thioredoxins and thioredoxin reductases [45,46]. Yap1 has both a nuclear localization sequence (NLS) and a nuclear export sequence (NES) where the export protein Crm1 binds. In unstressed yeast the cellular dynamics are such that, although Yap1 continually shuttles between the nucleus and the cytoplasm, it is predominantly located in the cytoplasm and Yap1-dependent genes are not transcribed [47,48]. Allicin directly oxidizes the Yap1 Cys598 and Cys620 residues in the C-terminal nuclear export sequence (NES), blocking the binding of the Crm1 export protein, thus allowing Yap1 to accumulate in the nucleus and to activate the expression of target OSR genes (Figure 1) [49]. Allicin-induced Yap1 activation also occurs in a ∆*gpx3* mutant. This confirms that activation is direct and independent of H_2_O_2_. At the moment it is unclear to what extent allicin’s oxidative activity might contribute to by any downstream production of reactive oxygen species (ROS) like H_2_O_2_ [49].

### 2.2. The Mammalian Keap1-Nrf2/ARE Oxidative Stress Sensor System

The OSR in mammalian cells is coordinated by the Keap1-Nrf2/ARE system which responds to reactive electrophilic species [50,51]. The Keap1 (Kelch-like ECH-associated protein) sensor protein has free cysteine thiols in its reduced state that are sensitive oxidation targets, making the oxidative stress sensor mechanism in mammals similar to that of Yap1 in yeast. In contrast to Yap1, which is itself a transcription factor, the reduced Keap1 oxidation sensor sequesters the Nrf2 transcription factor in a protein complex. In this complex Nrf2 is a substrate for ubiquitination and therefore degradation in the cytosol, making Nrf2 unavailable for transcription. However, when the redox-sensing cysteines in Keap1 are alkylated by electrophiles, Nrf2 dissociates from the complex and is no longer targeted for degradation (Figure 1). The Nrf2 transcriptional regulator, freed from the complex, migrates to the nucleus, associates with its co-transcription factor Maf, and facilitates the transcription of genes which have an antioxidant response element (ARE) in their promoters. The products of these ARE genes, which include antioxidative enzymes, giving rise to so-called phase II responses to oxidative stress [52]. Allicin, as a low *M*_r_ electrophilic oxidant is detected by, and activates, the Keap1-Nrf2/ARE system in mammalian cells [41,53].

Thiol oxidation chemistry is the common denominator employed in the OS-sensing systems of very different organisms (Figure 1). Taking advantage of this, we developed a very sensitive reporter system which can detect thiol-reactive low *M*_r_ electrophiles like allicin. We took the YKL071w gene (*Oxidative Stress Induced 1*, *OSI1*) from yeast that was reported to be the gene most highly induced by allicin [54] and fused its promoter to a luciferase reporter. The expression of *OSI1* is absolutely Yap1-dependent [49] making the *OSI1::luciferase* fusion a highly sensitive reporter of Yap1 activation in yeast. Comparing the yeast reporter system with an Nrf2-activated promoter-luciferase reporter system in mammalian cells showed congruence of effect. Moreover, the yeast-based system offers a lot of advantages over the human cell based assay, particularly in terms of sensitivity, the dynamic response, and ease of handling—making it advantageous in large scale screening procedures such as the detection of skin irritants or electrophilic activity of pharmaceuticals, reducing the need for animal testing [53].

### 2.3. Bacterial Redox Sensors

The OSR in bacteria is controlled by different redox sensitive regulators depending on the species. For example, in Gram-negative *Escherichia coli*, the transcriptional regulator OxyR has cysteine residues that can be directly oxidized by H_2_O_2_, leading to intramolecular disulfide formation between Cys199 and Cys208 in each subunit of the OxyR tetramer and subsequent activation of OxyR [55]. In Gram-positive firmicutes like *Bacillus subtilis* and *Staphylococcus aureus*, transcriptional repressors such as PerR are released from DNA by Fe-mediated oxidation of redox sensitive histidines, thus enabling the expression of genes involved in the H_2_O_2_ stress response [56,57,58]. Interestingly, genes that are controlled by these transcription factors were shown to be upregulated by allicin [31,59,60], perhaps indirectly by possible downstream H_2_O_2_ generation, or directly by oxidation of the structural Zn-binding cysteines that are necessary for PerR dimerization and DNA-binding. However, allicin can also directly alter the function of transcriptional regulators. For the redox-sensitive transcriptional repressors of *B. subtilis* like OhrR, YodB, HypR, and Spx, it was shown that their redox-sensitive Cys residues can be modified directly by allicin (Section 3.1., Table S1), thus connecting their regulon activity with an allicin-dependent mechanism for DNA release and derepression of response genes [60]. Numerous transcriptional regulators were shown to be targets of allicin stress either directly via demonstrated *S*-thioallylation, and/or indirectly by observing their regulon activity. In this way, it was shown that allicin stress in *S. aureus* induced the PerR, HypR, QsrR, MhqR, CstR, CtsR, HrcA and CymR regulons [59], and the OhrR, PerR, Spx, YodB, CatR, HypR, AdhR, HxlR, LexA, CymR, CtsR, and HrcA regulons were induced upon allicin stress in *B. subtilis* [60]. A description of these transcriptional regulators is given in supplementary Table S1. Several of these regulators are redox sensitive and are involved in the redox stress response and thiol homeostasis, or in stress responses that often occur simultaneously with oxidative stress, e.g., protein damage or metal stress. Like their eukaryotic counterparts in mammals (Nrf2/Keap1) and yeast (Yap1), these allicin-activated bacterial transcription regulators are important for the oxidative stress response.

### 2.4. The Central Role of GSH Metabolism in Allicin Resistance

**Figure 2:**
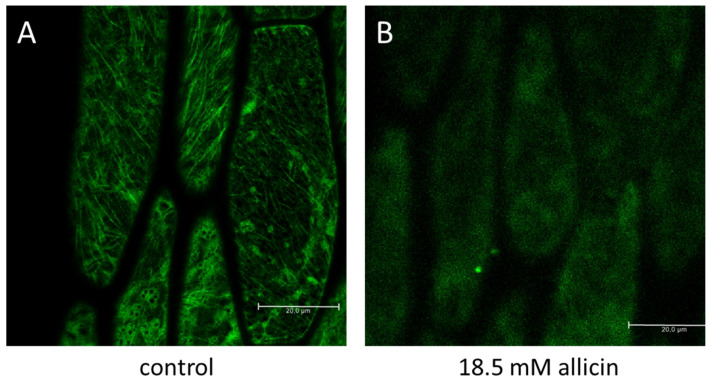
The influence of allicin on the organization of tubulin. Three-day-old *Arabidopsis* seedlings expressing GFP-tagged tubulin were incubated for 10 min in 18.5 mM allicin and subsequently viewed with a confocal laser scanning microscope (Leica, Wetzlar, Germany). While the tubulin filament structure is clearly visible in the water control (**A**) it takes on a more amorphous appearance after treatment with allicin (**B**). The scale bar = 20 µm.

Glutathione (γ-l-Glutamyl-l-cysteinylglycine, GSH) is present at up to 10 mM in the cytosol of many organisms and is recognized as a major cellular antioxidant that protects cells in a first line of defense against oxidative stress [39,61,62,63,64]. Although the total concentration of other free thiol groups in a cell can exceed that contributed by GSH, the latter is the most highly abundant LMW thiol in most eukaryotes and many Gram-negative bacteria. Because allicin is such a potent thiol trapping reagent, GSH is a major reactant for allicin in cells and is in direct competition for allicin with other less abundant thiols in individual proteins. Because of its molecular abundance, GSH can titrate out and thus protect cells against allicin. GSH is synthesized in two steps from cysteine by the enzymes γ-glutamylcysteine synthase (abbreviated to Gsh1 in yeast, GshA in bacteria, GSH1 in plants) and glutathione synthase (abbreviated to Gsh2 in yeast, GshB in bacteria, GSH2 in plants). Both glutathione disulfide (GSSG) and *S*-allylmercaptoglutathione (GSSA), the product formed when allicin reacts with GSH, are reduced back to GSH and GSH plus allylmercaptan, respectively by the NADPH-dependent enzyme glutathione reductase (abbreviated as Glr in yeast, Gor in bacteria, and GR in plants). Glutathione reductase is dependent upon NADPH as a reductant which is produced in the Pentose Phosphate Pathway (Figure 2A,B). A number of recent studies with mutants affected in GSH synthesis and metabolism in plants, fungi, and bacteria have reinforced the central role of GSH in enabling cells to withstand the oxidative stress caused by allicin treatment.

#### 2.4.1. Plants (*Arabidopsis thaliana*)

Allicin does not pass easily through the wax plates and cuticle layers of the leaf surface. However, if *Arabidopsis* leaf discs are floated on an allicin solution, which diffuses into the discs at the edges, oxidative stress occurs and chlorophyll bleaching is observed [65]. In contrast, young seedling roots are not covered by wax or cuticular layers and are more sensitive to allicin. Seedling roots are recognized as a suitable model to investigate the redox control of plant development [66,67] and similarly offer a good experimental system to study allicin effects on redox in plants. GSH is responsible for maintaining the root meristem and is important to enable root growth [68]. Treatment of plant roots with butathionine sulfoximine, a GSH biosynthesis inhibitor, led to a concentration-dependent suppression of root development (*ibid*), which phenocopied the *rml1* (root-meristem less) mutant. The *rml1* mutant, also known as *cad2* because it confers cadmium sensitivity, was shown to be unable to synthesize GSH due to a mutation in the *GSH1* gene [69]. Accordingly, root length in *Arabidopsis* correlates with the root glutathione content. Furthermore, root development depends not only on the total amount of GSH, but also on the redox state of the glutathione pool. Thus, the development of root hairs depends on the ratio of oxidized to reduced glutathione, i.e., the GSH half-cell redox status [70,71]. Allicin causes a concentration-dependent reduction in root growth because allicin oxidizes GSH directly to GSSA (and indirectly to GSSG), thus not only reducing the absolute GSH concentration but also by increasing the GSH half-cell redox potential. Whereas wild type roots are sensitive to allicin at 50 µM, root growth of GSH-deficient mutants was affected at 12.5 µM. We showed that other GSH-reacting thiosulfinates and thiosulfinate analogues also led to a considerable growth inhibition of *Arabidopsis* roots [19,72,73]. Furthermore, GSH-deficient *pad2* mutants of *Arabidopsis*, which contain only about 20% of the wild type levels of GSH, were shown to be more susceptible to allicin than wild type seedlings [19,72]. These results support the hypothesis that GSH abundance and the redox state of the GSH pool are important in protecting cells against the effects of allicin.

In addition to depleting the GSH pool, allicin also affects the plant cytoskeleton organization. Using transgenic *Arabidopsis* lines expressing GFP-tagged tubulin [74], it was shown that the tubulin cytoskeleton loses its filamentous organization and becomes more amorphous after allicin treatment (Figure 2). We suspect that the treatment may affect the auxin gradient in the root, perhaps by affecting turnover of the PIN auxin exporters, leading to a phenotype similar to that seen in *pin* mutants [71,75].

#### 2.4.2. Yeast (*Saccharomyces cerevisiae*)

**Figure 3:**
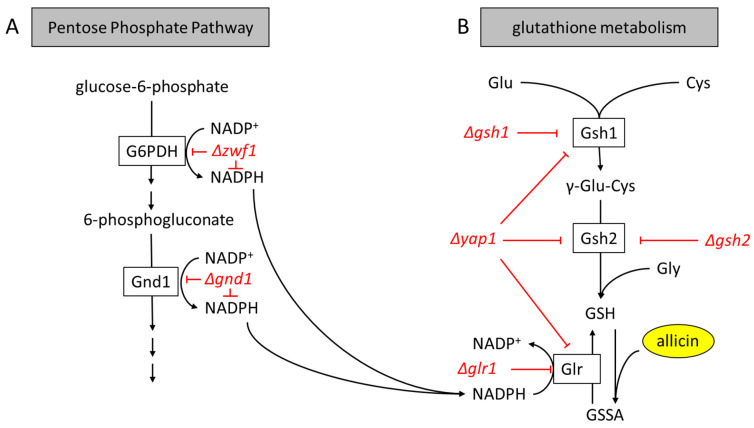
Summary of mutants in GSH synthesis and metabolism shown to affect allicin resistance. The diagram shows (**A**) the interplay between the NADPH-generating steps of the Pentose Phosphate Pathway and (**B**) GSH synthesis and metabolism. The steps at which the respective deletion mutants (*Δzwf1,* *Δgnd1,* *Δyap1*, *Δgsh1, Δgsh2,* *Δglr1*) act in yeast cells are shown in red. Allicin reacts directly with reduced glutathione (GSH) to form *S*-allylmercaptoglutathione (GSSA). The Pentose Phosphate Pathway provides NADPH as an electron donor for the enzyme glutathione reductase (Glr), that reduces GSSA to GSH and allylmercaptan (ASH, prop-2-ene-1-thiol). Yap1 is the central regulator for the oxidative stress response in yeast and activates several genes important for the antioxidant response, including *gsh1* and *gsh2* whose protein products catalyze GSH biosynthesis, and *glr1* which is necessary for the reduction of GSSG and GSSA to GSH by Glr1. Cysteine (Cys) and glutamine (Glu) are converted to γ-l-glutamyl-l-cysteine by Gsh1, and the addition of glycine (Gly) is catalyzed by Gsh2 to form GSH.

A chemogenetic screen of a yeast deletion mutant library revealed a group of mutants which showed hypersusceptibility to allicin compared to the wild type [49,72,76]. The mutants identified (Δzwf1, Δgnd1, Δyap1, Δgsh1, Δgsh2, Δglr1) are positioned in yeast metabolism in Figure 3A,B. All the mutants were either affected in their ability to synthesize GSH or to reduce GSSG. Yap1 is the oxidative stress activated transcription factor which transcribes GSH metabolism genes, including gsh1, the gene for the first committed and rate-limiting enzyme for GSH synthesis. Furthermore, gsh2 is also Yap1-dependent, as is glr1. Glutathione reductase (Glr1) reduces GSSG back to GSH and is dependent on the activities of glucose-6-phosphate dehydrogenase (G6PDH, encoded by the zwf1 gene) and 6-phosphogluconate dehydrogenase (Gnd1), respectively, to provide the necessary NADPH reducing equivalents from the Pentose Phosphate Pathway (Figure 3A,B). These results confirm the mode of action of allicin in targeting cellular thiols and show the major protective role of GSH in allicin-stressed cells.

#### 2.4.3. Glutathione in Gram-Negative Bacteria (*E. coli*, *Pseudomonas* spp.) and Bacillithiol in Gram-Positive Firmicutes (*B. subtilis*, *S. aureus*)

Like in eukaryotes, GSH in Gram-negative bacteria, and the functional analog bacillithiol (BSH) in Gram-positive firmicutes, are major allicin targets and key players when it comes to defense against allicin. Sublethal doses led to a significant decrease in GSH in *E. coli* from 2.6 mM to 1.7 mM [31]. In *S. aureus* a *bacilliredoxin* (Brx)-roGFP2-coupled redox biosensor demonstrated that allicin treatment led to a strong oxidative shift in the BSH redox potential [59]. Mutants unable to synthesize BSH were significantly more susceptible to allicin in both *B. subtilis* and *S. aureus* [59,60]. Transcriptomics data showed that genes required for BSH synthesis were upregulated 1.5- to 3-fold in *B. subtilis* upon sublethal allicin treatment and 2- to 5-fold in *S. aureus*. Other BSH recycling-related genes, like *ypdA* encoding bacillithiol disulfide (BSSB) reductase, were also shown to be upregulated by allicin and involved in the defense against allicin in *S. aureus* [59,60]. Furthermore, YpdA was shown to catalyze reduction of *S*-allylmercapto-BSH (BSSA) to recycle reduced BSH in vitro [59]. In a screen for allicin-tolerant bacteria we identified a *Pseudomonas* isolate which we named *Pseudomonas fluorescens* Allicin Resistant-1 (*Pf*AR-1). The strain and its resistance-features will be discussed in more detail in Section 3, however, the strain had three glutathione reductase (*gor*) genes compared to the single copy in the closely related but less tolerant *Pf*0-1 strain. The higher gene copy number reflected a 2-fold higher Gor enzyme activity in *Pf*AR-1 compared to *Pf*0-1 [77]. In the same study it was demonstrated that an *E. coli* Δ*gor* mutant, which showed an increased susceptibility to allicin compared to the wild type, could be complemented by expression of a heterologous *Pf*AR-1 *gor* gene. An analogous experiment was performed with a Δ*ypdA* mutant of *S. aureus*, whereby the higher allicin susceptibility was complemented by plasmid-based expression of the wild type *ypdA* gene [59]. In conclusion, LMW thiols GSH and BSH, together with their regenerating enzymes Gor and YpdA, are the first line of defense against allicin in bacteria, similar to all other biological kingdoms investigated so far.

## 3. Transcriptomic and Proteomic Studies

### 3.1. Effects of Allicin on the Transcriptome and Proteome in Bacteria

Apart from LMW thiols like GSH or BSH, the cellular thiol pool also contains proteins with thiol groups as targets for allicin. Using label-free MS/MS proteomic studies or the isotope-encoded affinity tag (ICAT) based OxICAT procedure, it was demonstrated that a broad spectrum of cellular proteins in *E. coli*, *Pseudomona*s, *B. subtilis*, and *S. aureus* was indeed *S*-thioallylated within minutes after sublethal allicin treatment [31,59,60]. The *S*-thioallyl proteomes of the Gram-positive bacteria *B. subtilis* and *S. aureus* were characterized by shotgun linear trap quadrupol (LTQ)-Orbitrap mass spectrometry of the tryptic peptides after treating the cells with allicin to identify peptides with a mass shift of 72 Da [59,60]. In *B. subtilis,* 79 proteins were identified after 30 min of allicin treatment and major targets were translation elongation factors, surfactin synthetase, antioxidant enzymes, redox-sensitive transcription factors, and biosynthetic enzymes for amino acids and nucleotides [60]. In *S. aureus,* 57 redox-sensitive proteins were found *S*-thioallylated, and the allylated proteins were functionally related to those allylated in *B. subtilis* [59,60]. In summary, detailed analysis of the targets revealed that allicin modified conserved redox-sensitive Cys residues in abundant cellular proteins as well as in lower abundant transcription factors in the proteomes of *S. aureus* and *B. subtilis* [59,60]. In this regard it is important to note that diallyl tetrasulfane reacted similarly with thiols and *S*-thioallylated a similar spectrum of proteins in *B. subtilis* to allicin [60].

The *S*-thioallylated allicin proteome has also been characterized in Gram-negative *E. coli* cells. Here again highly abundant cellular proteins were targeted, predominantly enzymes of primary metabolism, elongation factors, and ribosomal proteins [31]. Thus, interestingly, it seems that not only the accessibility/redox-sensitivity of a cysteine thiol makes it a preferred target for *S*-thioallylation but also the protein abundance within the cell. This likely reflects simple concentration-dependent competition between the reactants in a complex mixture and reinforces why GSH, which can be present at up to 10 mM intracellular concentration, is very effective at titrating out allicin and protecting cellular proteins against its action.

Upregulated transcripts and proteins, newly synthesized upon allicin treatment, reveal cellular responses to deal with allicin as well as indicating the damage it causes. Thus, a shared response to allicin in *E. coli*, *B. subtilis*, *S. aureus*, *P. aeruginosa,* and *P. fluorescens Pf*0-1 is the upregulation of transcripts and synthesis of proteins to alleviate the consequences of protein damage. For example, allicin (>1 mM, 15 min at 30 °C) caused aggregation of *E. coli* proteins in cell lysate. Assuming this effect also occurs in intact cells, it could be addressed by the observed allicin-induction of chaperones and proteases which are part of the heat shock response. Likewise, the CtsR and HrcA controlled regulons encoding Clp proteases and DnaK/GroESL chaperones were highly induced in the transcriptomes of *B. subtilis* and *S. aureus* [59,60]. Similarly, oxidative unfolding of proteins by allicin was countered by the induction of amino acid biosynthesis genes, ribosomal proteins, and translation factors in several bacteria tested [31,59,60,78,79]. It was shown that the ability to induce the heat shock response in *E. coli* was essential to overcome the effects of allicin stress. This was demonstrated with an *rpoH* mutant which was not able to induce the heat shock response, and unlike the wild type was unable to recover from allicin treatment [31]. Additionally, allicin also induced oxidative stress response genes controlled by the OxyR and PerR regulons, e.g., *katA*, *ahpCF,* and thioredoxins in *B. subtilis*, *E. coli,* and *S. aureus* [31,59,60,78].

An interesting effect of allicin highlighted by the data on upregulated transcripts or proteins, respectively, is damage of the cellular envelope. For example, in *S. aureus*, it was observed that allicin led to peptidoglycan fragmentation [80] and to the induction of the *GraS* cell wall stress regulon [60]. In *E. coli*, the outer membrane protein A (OmpA) and lipopolysaccharide protein P (Lpp), which are structural components in the cell envelope, were both induced after allicin treatment [31]. In *B. subtilis,* glucosamine-6-phosphate deaminase 2 (*nagBB*/*gamA*) and the oxidoreductase *dltE,* both responsible for cell wall restructuring via peptidoglycan degradation and teichoic acid modification, were also induced [78]. In *P. aeruginosa* alanine-tRNA can alter permeability in response to oxidative stress and membrane damage by aminoacylation of the phosphatidyl headgroups in the membrane. Thus, newly synthesized alanine-tRNA ligase might indicate involvement of cell envelope stress [78]. In addition to transcriptomic and proteomic studies, plasma membrane bubbles in the form of extrusions at the cell wall in *B. subtilis* were observed under the microscope after allicin treatment. This suggested that the plasma membrane might have extruded through holes in the cell wall created by allicin [78].

In addition to common responses to allicin, specific bacteria also showed unique responses. For example, although some aspects of the response of *P. aeruginosa*, e.g., the synthesis of various chaperones, matched the responses of other bacteria, Wüllner et al. [78] showed that *P. aeruginosa* does not synthesize additional proteins for increased antioxidants or other redox-related proteins upon sublethal allicin treatment. This might reflect the lesser effectiveness of allicin against *P. aeruginosa* PA01, which is seen in the much higher minimal inhibitory concentration (MIC ≥ 512 µg/mL allicin) compared to *E. coli* K12 (128 µg/mL allicin) and *B. subtilis* 168 (64 µg/mL allicin) [31,78]. Another so far unique aspect of allicin stress resistance was discovered in *S. aureus*. When Loi et al. investigated the allicin-induced changes in the transcriptome of *S. aureus*, they observed that the genes of the *hypR-merA* operon were strongly induced (195- to 228-fold change) [59]. It was already known that the transcription factor HypR was oxidized under strong oxidative and disulfide stress conditions, e.g., by hypochlorite or diamide stress, leading to de-repression of the *merA* gene encoding a flavin disulfide reductase. The NADPH-dependent MerA enzyme enabled increased survival of *S. aureus* during macrophage infection and hypochlorite stress [59], presumably by protecting *S. aureus* against the oxidative burst from activated macrophages [81]. The possible role of MerA in allicin protection was investigated in phenotype assays with *merA* mutants, which showed a decreased survival during allicin stress. In addition, purified MerA protein consumed NADPH when mixed with allicin, indicating that MerA was able to use allicin as a substrate [59]. Despite this specific resistance mechanism, allicin remains a potent, dose-dependent natural antimicrobial substance that is quite effective against methicillin resistant *S. aureus* (MRSA) [82].

Since allicin targets such a broad range of different molecules simultaneously within cells, spontaneous mutation to resistance is rarely observed in the laboratory [19]. We reasoned that we would most likely be able to find high allicin tolerance in nature in microorganisms living in close association with the garlic plant. We isolated and investigated a *P. fluorescens* isolate from garlic which showed an exceptionally high level of allicin resistance compared to other bacteria. We named this isolate *P. fluorescens* Allicin Resistant-1 (*Pf*AR-1) [77]. A *Pf*AR-1 genomic library was prepared and shotgun transferred into an allicin-sensitive *P. syringae* strain. Genomic clones which conferred resistance were found in a gain-of-function screen. Surprisingly, these clones were also able to confer allicin resistance to phylogenetically distant *E. coli* cells, suggesting a universal role in allicin protection for the respective genes. Genomic sequencing revealed that each genomic clone represented a small part (in average 10 kbp) of larger genomic regions present in three copies in the *Pf*AR-1 genome. GC content and codon usage indicated that the genomic regions were genomic islands (GIs) that had been acquired by horizontal gene transfer. Most of the genes which the clones had in common (*disulfide bond protein A* (*dsbA*), thioredoxin (*trx*), potassium/proton antiporter *KefC* (*kefC*), old yellow enzyme (*oye*), and alkyhydroperoxide D (*ahpD*)) were shown by transposon mutagenesis to be needed for allicin resistance because mutants showed increased sensitivity. However, over-expression of each gene separately showed that *ahpD* and *dsbA* were the only single genes able to confer allicin resistance to the allicin-susceptible isolates *P. syringae* 4612 or *P. syringae* 1448A. This suggests that the other genes work incrementally together to enhance tolerance to the effects of allicin. The resistance-conferring clones made up only a part of the total length of the GIs. The congruent sets of genes from the GIs outside of the resistance-conferring clones were highly sequence related and largely showed redox related functions. For example, a copy of glutathione reductase (*gor*) was present in two of the three repeats. This adds up to three copies of the *gor* gene in the *Pf*AR-1 genome. As mentioned above, the unusually high *gor* copy number and enzyme activity was shown to be linked to allicin resistance by genetic complementation studies [77]. In this way, *Pf*AR-1 exhibits multiple mechanisms of resistance to allicin encoded in several genes of varying major or minor incremental effect. This is an example of the evolutionary investment, involving gene duplication and horizontal gene transfer, which has enabled this bacterium to thrive under the strong selection pressure of its ecological niche.

A proteomic study showed that in non-stressed cells of *Pf*AR-1 and *Pf*0-1, cellular proteins were largely in a reduced state with only 76% and 77% respectively showing up to 20% cysteine oxidation. Comparing the degree of protein oxidation after allicin treatment showed that whereas 65% of cellular proteins remained less than 20% oxidized in *Pf*AR-1, in *Pf*0-1 this was only 50%, suggesting a better global protection machinery for proteins against oxidation by allicin in *Pf*AR-1 than in *Pf*0-1 [79]. This enhanced protective background is due to the function of the genes on the genomic islands gained by horizontal gene transfer by *Pf*AR-1 that are absent from *Pf*0-1.

One of the differentially *S*-thioallylated proteins in *Pf*0-1 but not in *Pf*AR-1 was DNA gyrase subunit A (GyrA). DNA gyrase is an essential bacterial enzyme and for this reason it is a target for *de novo* antibiotic development [79]. In unstressed cells the degree of Cys433 oxidation in GyrA was approximately 6% in both *Pf*AR-1 and *Pf*0-1. After allicin treatment the degree of Cys433 oxidation increased to approximately 56% in *Pf*0-1 compared to only 11% in *Pf*AR-1. Since DNA gyrase activity is essential for DNA replication it is likely that its protection from allicin oxidation is an important component of *Pf*AR-1 resistance. It was possible that either the *Pf*AR-1 GyrA was intrinsically less oxidizable by allicin, or that the *Pf*AR-1 genetic background protected it from *S*-thioallylation more effectively than the *Pf*0-1 background. Both *Pf*AR-1 and *Pf*0-1 GyrA amino acid sequences were highly conserved but the *Pf*AR-1 GyrA showed an additional internal stretch of 40 amino acids compared to *Pf*0-1 GyrA. It was speculated that this extra domain might alter the protein conformation and protect Cys433 from allicin access, which could have been responsible for an intrinsic allicin resistance of this enzyme. However, by reciprocal genomic replacement of the *gyrA* subunits between *Pf*AR-1 and *Pf*0-1, it was found that the allicin susceptibility of *Pf*0-1 cells with the *Pf*AR-1 *gyrA* gene replacement was not decreased but actually increased slightly (but not significantly), while *Pf*AR-1 cells with the *Pf*0-1 *gyrA* gene replacement remained allicin resistant. Because GyrA is essential for DNA replication in bacteria, this means that the *Pf*0-1 GyrA protein must have been protected from *S*-thioallylation in *Pf*AR-1 cells. Furthermore, *Pf*AR-1 GyrA enzyme activity was inhibited by allicin in vitro to a greater extent than *Pf*0-1 GyrA activity. In conclusion, this study showed that the *Pf*AR‑1 genetic background was most likely responsible for the protection of GyrA against *S*-thioallylation by allicin, rather than an intrinsic difference in the two GyrA sequences [79].

Significantly, the allicin-resistance-conferring genomic clones of *Pf*AR-1 did not confer general oxidative stress resistance. Thus, protection was not enhanced against either cumene hydroperoxide or H_2_O_2_. This indicates that the resistance mechanisms conferred by the genomic clones addressed specifically the types of stress caused by allicin, rather than ROS (reactive oxygen species) in general. This agrees with the hypothesis that the cost to the organism of maintaining multiple copies of the genomic islands in *Pf*AR-1 is due to selection pressure enabling it to exploit its ecological niche on garlic. Normally bacteria do not support large genomic repeats because of the danger of intragenomic homologous recombination leading to loss of intervening essential genetic material. DNA syntenic to the *Pf*AR-1 genomic repeats was also present in the genomes of other plant-associated bacteria—for example *P. syringae* pv. *tomato* DC3000 (one syntenic region) and *P. salomonii* ICMP14252 (two syntenic regions). Both species had elevated allicin resistance compared to related strains without the syntenic region [77]. Since *P. salomonii* is a garlic pathogen [83], one can conclude that the genes are needed to help it colonize its biological niche, while the presence of such a region in *Pst*. DC3000, that is pathogenic to plants from the Brassica family [84], remains a curiosity up to this point. The bacterium of origin for the horizontal gene transfer has not be identified so far. A plasmid-borne syntenic region was reported, however, in the onion pathogen *Pantoea ananatis.* This plasmid variant was shown to confer resistance against allicin, and it was necessary for efficient colonization of the onion host, even though onions do not produce allicin at all [21] and only relatively small amounts of other thiosulfinates [85,86].

It is interesting to note that there is some functional overlap between the genes that are induced in response to allicin stress in *E. coli*, *B. subtilis*, and *S. aureus* and the core set of genes from the allicin resistance-conferring regions in *Pf*AR-1, as well as genes not present on the genomic clones but in the remaining parts of the genomic islands. For example, members of the thioredoxin family as well as proteins belonging to the chaperone class can be found among the upregulated genes as well as in the genomic repeats. However, the grouping of triplicated, horizontally transferred genes in *Pf*AR-1 is quite distinctive, underlining the evolutionary matchmaking of the genome to the ecological niche of the garlic plant.

**Figure 4:**
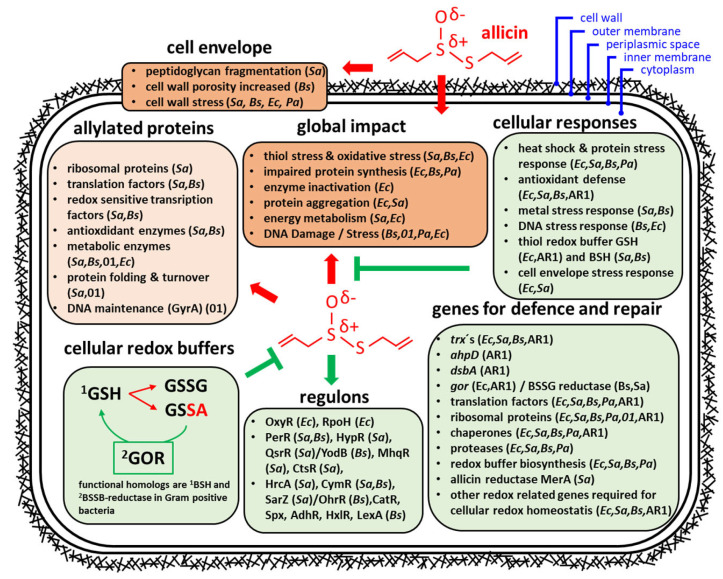
Summary of some of the effects of allicin on bacterial cells. The bacteria are: *Ec* = *E. coli* K12 [31,78], *Sa* = *S. aureus* USA300 [59,80,87], *Bs* = *Bacillus subtilis* strain 168 [60,78], *Pa* = *Pseudomonas aeruginosa* PA01 [78], 01 = *P. fluorescens* 01 [88], AR1 = *P. fluorescens* AR1 [77]. Abbreviations are: Trx = thioredoxin; AhpD = alkylhydroperoxidase D; DsbA = disulfide bond protein A; GSH = glutathione; GSSG = glutathione disulfide; GSSA = *S*-allylmercaptoglutathione; BSH = bacillithiol; BSSB = bacillithiol disulfide; Gor = glutathione disulfide reductase; GyrA = DNA gyrase subunit A. The heat and oxidative stress regulons induced under allicin stress in different bacteria are indicated, that are either regulated by *S*-thioallylation of the redox-sensitive regulators (YodB, OhrR, HypR, CatR) or by other mechanisms, such as increased oxidatively damaged proteins (e.g., RpoH, HrcA, CtsR). Background information about the functions of the transcription factors and their regulons is given in Table S1.

An overview of the genes relevant to allicin resistance found in studies with different bacteria is given in Figure 4 and Table S1.

### 3.2. Effects of Allicin on the Proteome of Human Cells (Jurkat T Lymphocytes)

**Figure 5:**
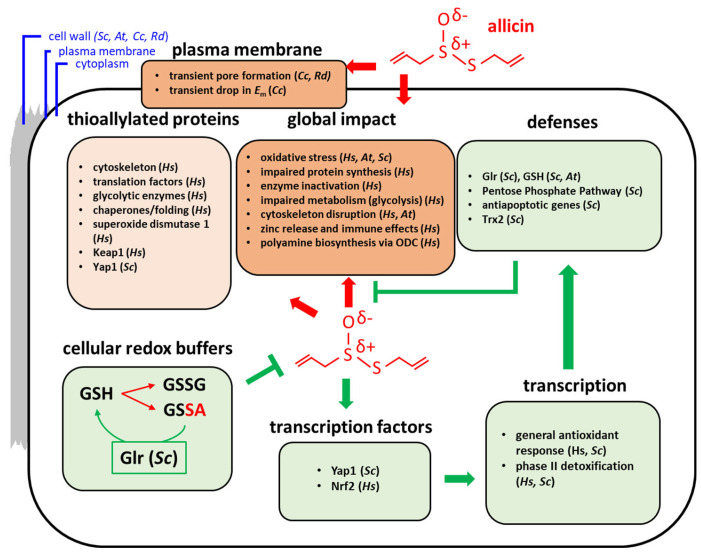
Summary of some of the effects of allicin on eukaryotic cells. Selected proteins are directly *S*-thioallylated and their biochemical function is thus altered (in most cases inhibition). This manifests itself in a general oxidative stress response, changes in metabolism and the cytoskeleton, an altered immune response and an influence on polyamine metabolism. A direct influence on cell membrane integrity can also be observed. At the same time, cells also respond to allicin exposure, for example through redox-sensitive transcription factors whose activity can be stimulated by allicin. Typical factors are the Nrf2-Keap1 system in mammals or the Yap1 transcription factor in yeast. These lead to the activation of protective mechanisms against oxidative stress. Various defense mechanisms are subsequently activated, with GSH synthesis being of particular importance. Associated with this is the Pentose Phosphate Pathway, in heterotrophic organisms the most important source of NADPH, the electron donor for glutathione reductase (Glr, GR). However, the expression of anti-apoptotic genes is also significant for protection against allicin, as is the phytohormone jasmonic acid in plants. Thioredoxins, especially Trx2, also seem to play a subordinate role, and their contribution is not as marked as that of the glutathione system. *At* = *Arabidopsis thaliana*, *Cc* = *Chara corallina*, *Hs* = *Homo sapiens*, *Rd* = *Rhoeo discolor*, Sc = *Saccharomyces cerevisiae* [24,49,53,63,75,88,90,92].

The Jurkat cell line was established from the blood of a patient with T cell leukemia in the late 1970s and the cells are widely used to study T-cell signaling and the effects of anti-cancer treatments [89]. Within 10 min of exposure to 100 µM allicin, 332 proteins were shown to have been *S*-thioallylated, including several which are recognized targets for cancer therapy [90]. This included 8 out of the 10 glycolytic enzymes, including enolase, and several cytoskeletal proteins. Again, as with bacterial cells, many of the target proteins were highly abundant cellular proteins, for example the cytoskeletal proteins tubulin, actin, cofilin, filamin, and the actin-binding protein plastin-2, as well as heat shock chaperones (Figure 5) [90]. This again emphasizes the somewhat indiscriminate nature of allicin’s action in targeting accessible, oxidizable thiols per se in cells and the law of mass action meaning that abundant molecular species have a competitive advantage over quantitatively minor species. The 100 µM allicin concentration used in the above study was ‘biocompatible’, that is to say the treated cells showed no reduction in metabolic activity over a 24 h period compared to untreated controls. Nevertheless, after 10 min exposure to 100 µM allicin the cells showed an approximately 50% decrease in free thiols. This early time point was chosen to identify primary allicin targets rather than later protein modifications due to cell adaptation to allicin stress [90]. The efficacy of allicin as a thiol trapping reagent is shown clearly in these experiments, highlighting the potential for allicin to influence cell physiology at sublethal, biocompatible doses. In this context see Section 4 for a consideration of the bioavailability of allicin in the body. Furthermore, it is important to note that different cell lines have been shown to respond in certain ways in common, but in other ways differently to allicin [91].

## 4. Allicin in Medicine

There is a plethora of information in the literature, based mainly on studies with cell cultures, attesting to the potential positive effects of allicin, not only in relation to several diseases, but also on the immune system. Thus, allicin at sublethal doses was reported to reduce NO production in macrophages by inhibiting inducible NO-synthase (iNOS), and furthermore, by increasing the cytosolic [Zn^2+^] to stimulate the immune response in general [90,92]. Furthermore, there is a wealth of ancient and modern anecdotal material regarding the beneficial health effects of garlic consumption. However, evidence-based scientific studies, especially those including rigorous double blind, statistically adequate, independently funded, clinical studies are generally lacking. Nowhere has the conundrum of the promise that allicin offers and the difficulties associated with its use, been better reviewed than in Chapter 5 of the excellent book ‘Garlic and Other Alliums’, and the reader is referred to this text for a detailed treatment of numerous published studies [21]. In the opinion of the present authors, the potential for allicin, and for that matter some of the other sulfur-containing compounds found in Alliums, to be developed for therapeutic uses is real. A major problem that needs to be solved in order to move to the next stage for that potential to be realized is to develop a delivery system in the body that can achieve a therapeutically relevant concentration in the target tissues without damaging non-target cells. Much more fundamental research is necessary before allicin might be used safely in treating diseases.

In essence two fundamentally different therapeutic situations exist. One would be to use allicin’s antimicrobial activity against infection and the other relates to allicin’s physiological effects on body cells in combatting non-infectious conditions, such as coronary disease and cancer.

Before looking in detail at specific examples, it is useful to consider the fate of allicin in the body, a topic that is relevant to its bioavailability. Cooking garlic quickly destroys any allicin. On heating, allicin spontaneously decomposes to 2-propenesulfenic acid and 2-propenethial (thioacrolein) which react further to produce alkyl disulfides, polysulfanes, vinyl dithiins, and ajoene [21,22]. Many of these compounds are physiologically active in their own right, but when considering garlic in the diet as a source of allicin, only raw garlic counts. Under the acidic conditions in the stomach allicin hydrolyzes rapidly to 2-propenethiol (PT), which is metabolized to allyl methyl sulfide (AMS). Both PT and AMS are excreted in the urine and are recognizable in the unpleasant “garlic breath”, which often makes consumers of garlic unpopular amongst those who have not similarly participated [93,94,95,96]. These reactions reduce the amount of allicin available to react with cellular thiols. Nevertheless, organisms are exposed to allicin in varying amounts in different situations, not least via the pulmonary route, and any allicin entering cells can have profound effects on cellular metabolism.

**Figure 6:**
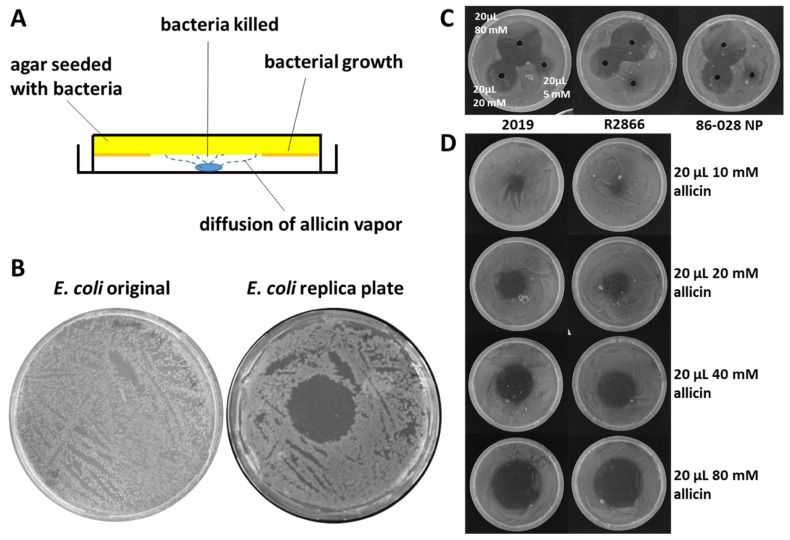
Inhibition of bacteria by allicin vapor and solutions. (**A**) Bacteria-seeded agar in a Petri plate inverted over a drop of allicin solution in the lid prevents the growth of the bacteria and leads to a clear zone after incubation. (**B**) In this variation of the test the inoculum was spread over the surface of agar medium with a Drigalski spatula and allowed to grow overnight at 37 °C to make a lawn on the surface before inverting the plate over a droplet of allicin solution. A replica plate was then prepared from the lawn using a sterile velvet pad and this showed that the growth of bacteria was not only prevented by allicin vapor, but that bacteria in a pre-existing lawn can also be killed outright. (**C**) Agar diffusion test showing the inhibition of the growth of three strains of lung pathogenic *Haemophilus influenzae* (strains 2019, R2866, and 86-028 NP) spread over the surface of agar medium with a Drigalski spatula. Allicin solution (20 µL) at 5, 20 or 80 mM was pipetted into wells in the bacteria-seeded agar. (**D**) Agar plates of the 2019 and R2866 *H. influenzae* strains were inverted over a 20 µL droplet of allicin solution at 10, 20 or 80 mM (absolute amounts = 32.5, 65, 130, and 260 µg allicin, respectively). Plates were incubated for 17 h at 37 °C.

Allicin taken up by cells, or entering the bloodstream, reacts readily with GSH in competition with cysteine residues in proteins. For this reason, it is difficult to envisage being able to achieve a therapeutically relevant concentration of allicin in cells anywhere in the body by simply swallowing it. Furthermore, as mentioned in the introduction, allicin is an irritant which stimulates pain-sensing neurons, and self-medication has led to a spate of reports of self-inflicted harm [21]. Thus, while small amounts are prized in culinary contexts, over-exposure to allicin is clearly harmful. Having issued that precautionary note, it is also true that there is a lack of volatile antibiotics available for use in medicine, and this possibility for allicin deserves further exploration [88,97,98,99]. Perhaps, after further carefully controlled research and all necessary precautions, a pulmonary exposure route for allicin vapor might be able to be developed under appropriate circumstances to achieve therapeutically effective concentrations in the lungs. A simple experiment to demonstrate the effectivity of allicin vapor against microorganisms is shown in Figure 6A,B. The example with *E. coli* shows that allicin vapor does not just exert bacteriostatic effects to prevent growth, but can also actively kill cells, i.e., there is a clear bactericidal effect of the vapor. The lawn of *E. coli* on the left Petri plate was exposed to allicin vapor and then replica-plated using a sterile velvet pad to pick up inoculum from the existing colonies and transfer it to a new plate. The pattern of cells on the original plate was replicated onto the second plate—shown on the right in Figure 6B. It can be seen that no living *E. coli* cells were transferred from the central region of the original plate where the cells were killed by the allicin vapor, and were therefore no longer able to grow even in the absence of allicin.

### 4.1. Allicin Activity against Respiratory Tract Pathogens

Because allicin vapor is bactericidal, the idea to use it to combat lung infections is attractive. Indeed, there is a precedent for this in a historical report from the pre-antibiotic-era [97]. Several patients with pulmonary tuberculosis were reported to have been successfully treated by inhaling the vapor from garlic preparations. Patients were fitted with a face mask with a pouch containing juice from pulverized garlic mixed with ethanol and eucalyptus oil to help to mask the smell. They breathed in the fumes for two one-hour periods daily, and a good success rate against pulmonary TB was reported [97]. Interestingly, more recent experiments have suggested that the allicin and ethanol vapors work together synergistically to kill microbes, which was probably unknown to Dr. Minchin at the time [98].

Antibiotics are usually taken orally or injected as solutions, whereas gas-phase antibiotics administered via the pulmonary route are not generally known. A recent survey showed that several lung-pathogenic bacteria, including MDR strains, could be inhibited in vitro by allicin vapor and the aerodynamic behavior of allicin aerosols and vapor has been investigated in a lung model, thus allowing some preliminary determination of dosage and synergy without the need for animal experiments [88,98,99]. Nevertheless, progression to the next stage will require carefully controlled, ethically sound animal experiments before therapeutic protocols for humans can become feasible.

An example showing that allicin can inhibit the growth of the opportunistic lung-pathogenic bacterium *Haemophilus influenzae* in a straightforward agar plate diffusion assay, and importantly via the gas phase in the inverted Petri plate assay, is illustrated in Figure 6C,D. The size of the inhibition zones shows a clear dose-dependent relationship to allicin exposure. To our knowledge allicin has not previously been reported to be active against *H. influenzae*, which is recalcitrant to amoxicillin and ampicillin and resistance to β-lactam antibiotics specifically, and is rising strongly in Nontypeable *H. influenzae* (NTHi) isolates.

Currently, the world is in the grip of a COVID-19 pandemic caused by the SARS-CoV-2 virus. Cysteine-containing proteins of this respiratory tract-infecting virus may be a direct target for *S*-thioallylation, either in the extracellular free virus particle or during replication in host cells. At present this remains unclear, however, there is a report using in silico analysis that the SARS-CoV-2 main protease may be a target for *S*-thioallylation [100]. Aside from this in silico speculation, experimental data suggest that garlic may be able to have a beneficial effect on helping to prevent severe COVID-19 via stimulation of the immune system by increasing intracellular Zn^2+^ levels [90]. Thus, it was shown that at biocompatible doses, allicin stimulated the interleukin-1 (IL-1) dependent release of IL-2 by mouse EL-4 T-cells. This effect is zinc dependent and allicin increases [Zn^2+^]cyt by causing intracellular Zn^2+^ release from proteins by oxidizing the Cys residues which chelate it, e.g., from SOD-1, shown to be a major target for *S*-thioallylation in human Jurkat cells [90]. The importance of Zn^2+^ for the immune system in relation to preventing severe COVID-19 has recently been reviewed [101].

### 4.2. Allicin *vs.* Cancer

It has been reported many times that allicin has pro-apoptotic and antitumorigenic properties against several cancer types, mostly in studies using cultured cells [41,91,102,103,104,105,106,107,108,109,110]. However, osteosarcoma and cholangiosarcoma in mice have for example been reported to respond to intraperitoneal injection of allicin in vivo [103,108].

As mentioned above, allicin has multiple cellular targets in human cells, including for example cytoskeletal proteins and glycolytic enzymes that are also candidates for cancer treatments [90]. Several studies have shown that signaling pathways important in tumor cell lines can be interfered with by allicin treatment [41,102,103,104,105,106]. Furthermore, synergism between allicin and some anticancer drugs, e.g., cyclophosphamidum, artesunate, and all-trans retinoic acid (ATRA) has been demonstrated [107,108,109]. It is also important to note that high GSH levels are associated with many tumor types. High GSH protects the tumor cells against oxidative stress and interferes with the activity of some therapeutic agents [111,112]. In this regard, allicin’s activity as a thiol trapping reagent that readily reacts with and depletes the GSH pool may be very helpful in combating tumor cells. Indeed, strategies to lower GSH levels have been combined with tumor therapies, for example the GSH synthesis inhibitor buthionine sulfoximine (BSO) was used in conjunction with the electrophile melphalan to treat neuroblastoma [113,114]. Another example is the use of BSO with azathioprine to treat human hepatocarcinoma and colon carcinoma [115]. Thus, the broad-ranging effects of allicin in cells could be advantageous rather than disadvantageous when it comes to cancer treatments. In addition to the documented effects of allicin on the cytoskeleton which directly affect cell division, and glycolytic enzymes relevant to the Warburg effect, a recently published study showed that allicin was 23,000 times more effective at inhibiting the enzyme ornithine decarboxylase (ODC) than was α-difluoromethylornithine (DFMO) [110]. ODC is especially important for neuroblastoma cell proliferation and DFMO is being tested in phase I/II human neuroblastoma trials with encouraging results. If a specific delivery system can be found, the potential advantages of allicin for treating neuroblastoma are obvious, as it not only targets ODC but concomitantly reduces GSH levels.

In this regard, the major problem of targeting allicin to tumor tissues (while leaving surrounding healthy cells unstressed) urgently needs to be overcome, and there is a need in the future for carefully controlled studies in animal models and clinical trials involving humans [21].

## Data Availability

Not applicable.

## Supplementary Materials

The following are available online. Table S1. Overview of the characteristics of prokaryotic transcriptional regulators whose activities were shown to be influenced by allicin treatment.
