# Supplementary material for: Allicin, the Odor of Freshly Crushed Garlic: A Review of Recent Progress in Understanding Allicin’s Effects on Cells
Source: Molecules. 2021 Mar 10;26(6):1505. doi: 10.3390/molecules26061505 (PMC8001868; doi:10.3390/molecules26061505)
Supplement: Supplementary file 1 [file molecules-26-01505-s001.pdf]

Table S1. Overview of the characteristics of prokaryotic transcriptional regulators whose activities were shown to be influenced by allicin treatment.

organism	regulator	characteristics	effect of allicin
Staphylococcus aureus USA300	PerR	• functions analogously to PerR in *Bacillus* (transcriptional repressor with redox-sensitive His-residues, see below) - controls *catalase*, *alkyl hydroperoxide reductase* and *thiol peroxidase*, *thioredoxin reductase*, and *mgrA* for the H_2O_2 stress response [1,2]	upregulation of regulon genes [3]
Staphylococcus aureus USA300	HypR	• transcriptional repressor whose repression is inhibited by oxidation of redox sensitive Cys residues (Cys33-Cys99 intersubunit disulfide bond formation) [4]<br>• responds to disulfide stress but not significantly to H_2O_2 stress and electrophilic species such as aldehydes [4]<br>• controls the disulfide reductase *merA* [4]	upregulation of regulon genes [3]
Staphylococcus aureus USA300	QsrR	• YodB family transcriptional repressor, which is released from DNA by Cys-*S*-quinonization (Cys5) [5]<br>• involved in quinone detoxification [5]. Quinones are electron carriers with antimicrobial and toxic properties (electrophilic oxidants) [6]	upregulation of regulon genes [3]
Staphylococcus aureus USA300	MhqR	• transcriptional quinone-sensing repressor [7]<br>• Cys residue does not contribute to DNA binding ability / no thiol based DNA interaction mechanism [7]<br>• confers resistance to quinone and to quinone-like antimicrobial compounds [7]<br>• does not respond to ROS, hypochlorous acid or aldehydes [7]	upregulation of regulon genes [3]
Staphylococcus aureus USA300	CtsR	• transcriptional repressor [8]<br>• controls the chaperones *dnaK* and *groESL* together with HrcA [8]<br>• also involved in response to sulfide toxicity via *cst* operon (Copper-sensing operon repressor (CsoR)-like sulfur-transferase repressor) [9]<br>• polysulfide or persulfide sensing repressor, CstR derivatizes with tetrasulfide or glutathione persulfide, leading to polysulfide bonds and to the release from DNA [9]<br>• Cys31 and Cys60 are required for regulatory activity [9]	upregulation of regulon genes [3]
Staphylococcus aureus USA300	HrcA	• transcriptional repressor of the heat shock response together with CtsR [8]<br>• contains no Cys residues (GenBank: AIO21276.1)<br>• controls *dnaK* and *groESL* chaperones together with CtsR [8]	upregulation of regulon genes [3]
Staphylococcus aureus USA300	CymR	• transcriptional repressor which is released from DNA by Cys-oxidation to Cys25-SOH [10]<br>• takes part in oxidative stress resistance [10]<br>• master regulator of cysteine metabolism [11]<br>• modulates biofilm accumulation [11]	upregulation of regulon genes [3]
Staphylococcus aureus USA300	SarZ (MgrH1)	• transcriptional repressor [12]<br>• released from DNA by oxidation of Cys13 residue [12]<br>• involved in peroxide stress defense, metabolic switching, antibiotic resistance, virulence and cell wall remodeling [12]	upregulation of regulon genes [3]
Staphylococcus aureus USA300	CsoR	• transcriptional regulator of copper resistance genes [13]	upregulation of regulon genes [3]
Staphylococcus aureus USA300	Zur	• Zn-responsive transcriptional repressor [14]<br>• contains six Cys residues (GenBank: CAC6155577.1)<br>• regulates staphylopine biosynthesis (siderophore for metal binding) and trafficking [15,16]	upregulation of regulon genes [3]
Staphylococcus aureus USA300	GraRS	• multi component regulatory system in the cell membrane [17]<br>• regulates cell wall remodeling for cationic antimicrobial peptide- and glycopeptide-resistance, e.g. against vancomycin [18]	upregulation of regulon genes [3]

		• involved in biofilm formation [19,20]<br>• required for growth at high temperatures and for oxidative stress resistance (Paraquat, H_2O_2) [21]	
Bacillus subtilis 168	PerR	• negative transcriptional regulator bound to DNA in unstressed conditions and released from DNA by oxidation of either His37 or His 91 or both residues by hydrogen peroxide, mediated via a coordinated Fe in PerR [22]<br>• Possesses four Cys residues for Zn coordination which is required for PerR dimerization and DNA binding [23]<br>• involved in oxidative stress response, regulates *ahpCF*, *katA*, *mrgA*, and heme biosynthesis genes [22]. *mgrA* is a Dps like DNA binding protein which can protect DNA against oxidative damage [24]	upregulation of regulon genes [25]
Bacillus subtilis 168	YodB	• transcriptional repressor, regulated by redox-sensitive Cys6, Cys101 and Cys108 [26,27]<br>• disulfide bond formation between Cys6 and Cys101 or Cys108 upon diamide stress [26]<br>• binding to DNA is inhibited after oxidation by thiol reactive compounds [27]<br>• involved in resistance to catechol and methylhydroquinone [27]	upregulation of regulon genes and YodB Cys101 was thioallylated by allicin [25]
Bacillus subtilis 168	OhrR	• involved in resistance to organic hydroperoxides [22]<br>• transcriptional redox sensitive repressor [22]<br>• MarR (multiple antibiotic resistance regulator) regulator [22]<br>• DNA-binding activity is regulated via Cys15 residue of OhrR [22]	*B. subtilis* Δ*ohrA* showed increased susceptibility to allicin. OhrA belongs to the OhrR regulon and encodes a peroxiredoxin. Additionally, OhrR Cys15 is thioallylated by allicin [25]
Bacillus subtilis 168	HypR	• redox sensitive transcriptional repressor [28]<br>• involved in disulfide stress resistance, e.g. against diamide [28]<br>• repression is inactivated by oxidation of Cys residues (Cys14-Cys49 disulfide bond formation) [28]	*B. subtilis* ΔhypR showed increased susceptibility to allicin and HypR Cys14 is thioallylated by allicin [25]
Bacillus subtilis 168	Spx	• activated by diamide / thiol stress [29]<br>• regulates bacillothiol (BSH) biosynthesis genes, the central redox buffer in Gram negative bacteria [30]<br>• activated via oxidation of redox sensitive Cys10 and Cys13 to form intramolecular disulfide bonds [29]<br>• binds to RNA polymerase under oxidative stress conditions [29,31]<br>• required for trx transcription [29,31,32]	regulon (e.g. thioredoxins or methionine sulfoxide reductases, BSH biosynthesis genes) were upregulated by allicin and *B. subtilis* Δspx showed increased susceptibility to allicin [25]
Bacillus subtilis 168	AdhR	• probably involved in reduction of aldehydes and in repair or degradation of thiol damaged proteins [33]<br>• Cys52 is required for AdhR activation [33]<br>• regulon confers resistance to formaldehyde [33]	upregulation of regulon genes [25]
Bacillus subtilis 168	HxlR	• transcriptional regulator which is required for the transcription of two key-enzymes of the monophosphate ribulose pathway, 3-hexulose-6-phosphate synthase and 6-phospho-3-hexuloisomerase, which are both involved in formaldehyde fixation [34,35]<br>• contains one Cys residue (UniProtKB - P42406)<br>• regulon confers resistance to formaldehyde [33,35]	upregulation of regulon genes [25]

Bacillus subtilis 168	LexA	• amino acid sequence does not contain any Cys residues that could react with allicin (UniProtKB - P31080)<br>• repressor of DNA damage SOS response [36,37]<br>• Arg49 and His46 are essential for operator binding and Lys53 and Ala48 are involved in (but not essential for) operator recognition [37]	upregulation of regulon genes [25]
Bacillus subtilis 168	CatR	• regulates catDE operon for catechol detoxification together with but independently from YodB [38]<br>• transcriptional redox sensitive repressor which binds to DNA via Cys7 [38], forms quinone induced intramolecular Cys7-Cys101 disulfide [26]<br>• involved in diamide and quinone stress response [26,27,38]	regulon induced by allicin and catR Cys7 was shown to be thioallylated by allicin [25]
Escherichia coli K12	OxyR	• transcriptional regulator which is activated by oxidation of a Cys residue via H_2O_2 to subsequently form an intramolecular disulfide bond with another Cys (Cys199-Cys208) [39,40]<br>• involved in oxidative stress response to alleviate H_2O_2 stress, e.g. by upregulation of AhpCF and KatG [41]	genes of the OxyR regulon were induced after allicin treatment, shown by newly synthesized proteins [42]
Escherichia coli K12	RpoH	• σ32 RNA-Polymerase factor [43]<br>• responsible for heat shock induction in *E. coli*, inducing chaperones and proteases [43]<br>• σ32 protein stability and its RNA-polymerase binding activity are negatively regulated by FtsH protease and DnaK and DnaJ chaperones [43]	genes of the RpoH regulon were induced after allicin treatment (newly synthesized proteins); RpoH protein half life increased after allicin treatment and a rpoH mutant was unable to recover from sublethal allicin stress [42]

References

1. Horsburgh, M.J.; Clements, M.O.; Crossley, H.; Ingham, E.; Foster, S.J. PerR Controls Oxidative Stress Resistance and Iron Storage Proteins and Is Required for Virulence in *Staphylococcus aureus*. *Infect. Immun.* **2001**, *69*, 3744, doi:10.1128/IAI.69.6.3744-3754.2001.
2. Ji, C.-J.; Kim, J.-H.; Won, Y.-B.; Lee, Y.-E.; Choi, T.-W.; Ju, S.-Y.; Youn, H.; Helmann, J.D.; Lee, J.-W. *Staphylococcus aureus* PerR Is a Hypersensitive Hydrogen Peroxide Sensor Using Iron-Mediated Histidine Oxidation *. *J. Biol. Chem.* **2015**, *290*, 20374–20386, doi:10.1074/jbc.M115.664961.
3. Loi, V.V.; Huyen, N.T.T.; Busche, T.; Tung, Q.N.; Gruhlke, M.C.H.; Kalinowski, J.; Bernhardt, J.; Slusarenko, A.J.; Antelmann, H. *Staphylococcus aureus* Responds to Allicin by Global *S*-Thioallylation – Role of the Brx/BSH/YpdA Pathway and the Disulfide Reductase MerA to Overcome Allicin Stress. *Free Radic. Biol. Med.* **2019**, *139*, 55–69, doi:10.1016/j.freeradbiomed.2019.05.018.
4. Loi, V.V.; Busche, T.; Tedin, K.; Bernhardt, J.; Wollenhaupt, J.; Huyen, N.T.T.; Weise, C.; Kalinowski, J.; Wahl, M.C.; Fulde, M.; et al. Redox-Sensing Under Hypochlorite Stress and Infection Conditions by the Rrf2-Family Repressor HypR in *Staphylococcus aureus*. *Antioxid. Redox Signal.* **2018**, *29*, 615–636, doi:10.1089/ars.2017.7354.
5. Ji, Q.; Zhang, L.; Jones, M.B.; Sun, F.; Deng, X.; Liang, H.; Cho, H.; Brugarolas, P.; Gao, Y.N.; Peterson, S.N.; Lan, L.; Bae, T.; He, C. Molecular Mechanism of Quinone Signaling Mediated through S-Quinonization of a YodB Family Repressor QsrR. *Proc. Natl. Acad. Sci. USA* **2013**, *110*, 5010, doi:10.1073/pnas.1219446110.
6. Monks, T.J.; Hanzlik, R.P.; Cohen, G.M.; Ross, D.; Graham, D.G. Quinone Chemistry and Toxicity. *Tox. Appl. Pharmacol.* **1992**, *112*, 2–16, doi:10.1016/0041-008X(92)90273-U.
7. Fritsch, V.N.; Loi, V.V.; Busche, T.; Sommer, A.; Tedin, K.; Nürnberg, D.J.; Kalinowski, J.; Bernhardt, J.; Fulde, M.; Antelmann, H. The MarR-Type Repressor MhqR Confers Quinone and Antimicrobial Resistance in *Staphylococcus aureus*. *Antioxid. Redox Signal.* **2019**, *31*, 1235–1252, doi:10.1089/ars.2019.7750.
8. Chastanet, A.; Fert, J.; Msadek, T. Comparative Genomics Reveal Novel Heat Shock Regulatory Mechanisms in *Staphylococcus aureus* and Other Gram-Positive Bacteria. *Mol. Microbiol.* **2003**, *47*, 1061–1073, doi:10.1046/j.1365-2958.2003.03355.x.
9. Luebke, J.L.; Shen, J.; Bruce, K.E.; Kehl-Fie, T.E.; Peng, H.; Skaar, E.P.; Giedroc, D.P. The CsoR-like Sulfurtransferase Repressor (CstR) Is a Persulfide Sensor in *Staphylococcus aureus*. *Mol. Microbiol.* **2014**, *94*, 1343–1360, doi:10.1111/mmi.12835.
10. Ji, Q.; Zhang, L.; Sun, F.; Deng, X.; Liang, H.; Bae, T.; He, C. *Staphylococcus aureus* CymR Is a New Thiol-Based Oxidation-Sensing Regulator of Stress Resistance and Oxidative Response *. *J. Biol. Chem.* **2012**, *287*, 21102–21109, doi:10.1074/jbc.M112.359737.
11. Soutourina, O.; Poupel, O.; Coppée, J.-Y.; Danchin, A.; Msadek, T.; Martin-Verstraete, I. CymR, the Master Regulator of Cysteine Metabolism in *Staphylococcus aureus*, Controls Host Sulphur Source Utilization and Plays a Role in Biofilm Formation. *Mol. Microbiol.* **2009**, *73*, 194–211, doi:10.1111/j.1365-2958.2009.06760.x.
12. Chen, P.R.; Nishida, S.; Poor, C.B.; Cheng, A.; Bae, T.; Kuechenmeister, L.; Dunman, P.M.; Missiakas, D.; He, C. A New Oxidative Sensing and Regulation Pathway Mediated by the MgrA Homologue SarZ in *Staphylococcus aureus*. *Mol. Microbiol.* **2009**, *71*, 198–211, doi:10.1111/j.1365-2958.2008.06518.x.
13. Baker, J.; Sengupta, M.; Jayaswal, R.K.; Morrissey, J.A. The *Staphylococcus aureus* CsoR Regulates Both Chromosomal and Plasmid-Encoded Copper Resistance Mechanisms. *Environ. Microbiol.* **2011**, *13*, 2495–2507, doi:10.1111/j.1462-2920.2011.02522.x.
14. Lindsay, J.A.; Foster, S.J. Zur: A Zn2+-Responsive Regulatory Element of *Staphylococcus aureus*. *Microbiology* **2001**, *147*, 1259–1266.
15. Fojcik, C.; Arnoux, P.; Ouerdane, L.; Aigle, M.; Alfonsi, L.; Borezée-Durant, E. Independent and Cooperative Regulation of Staphylopine Biosynthesis and Trafficking by Fur and Zur. *Mol. Microbiol.* **2018**, *108*, 159–177, doi:10.1111/mmi.13927.
16. Grim, K.P.; San Francisco, B.; Radin, J.N.; Brazel, E.B.; Kelliher, J.L.; Párraga Solórzano, P.K.; Kim, P.C.; McDevitt, C.A.; Kehl-Fie, T.E. The Metallophore Staphylopine Enables *Staphylococcus aureus*To Compete with the Host for Zinc and Overcome Nutritional Immunity. *mBio* **2017**, *8*, e01281-17, doi:10.1128/mBio.01281-17.
17. Li, M.; Cha, D.J.; Lai, Y.; Villaruz, A.E.; Sturdevant, D.E.; Otto, M. The Antimicrobial Peptide-Sensing System Aps of *Staphylococcus aureus*. *Mol. Microbiol.* **2007**, *66*, 1136–1147, doi:10.1111/j.1365-2958.2007.05986.x.
18. Meehl, M.; Herbert, S.; Götz, F.; Cheung, A. Interaction of the GraRS Two-Component System with the VraFG ABC Transporter To Support Vancomycin-Intermediate Resistance in *Staphylococcus aureus*. *Antimicrob. Agents Chemother.* **2007**, *51*, 2679, doi:10.1128/AAC.00209-07.
19. Boles, B.R.; Thoendel, M.; Roth, A.J.; Horswill, A.R. Identification of Genes Involved in Polysaccharide-Independent *Staphylococcus aureus* Biofilm Formation. *PLOS ONE* **2010**, *5*, e10146, doi:10.1371/journal.pone.0010146.
20. Shanks, R.M.Q.; Meehl, M.A.; Brothers, K.M.; Martinez, R.M.; Donegan, N.P.; Graber, M.L.; Cheung, A.L.; O'Toole, G.A. Genetic Evidence for an Alternative Citrate-Dependent Biofilm Formation Pathway in

Staphylococcus aureus That Is Dependent on Fibronectin Binding Proteins and the GraRS Two-Component Regulatory System. *Infect. Immun.* **2008**, *76*, 2469, doi:10.1128/IAI.01370-07.
21. Falord, M.; Mäder, U.; Hiron, A.; Débarbouillé, M.; Msadek, T. Investigation of the *Staphylococcus aureus* GraSR Regulon Reveals Novel Links to Virulence, Stress Response and Cell Wall Signal Transduction Pathways. *PLOS ONE* **2011**, *6*, e21323, doi:10.1371/journal.pone.0021323.
22. Zuber, P. Management of Oxidative Stress in *Bacillus*. *Annu. Rev. Microbiol.* **2009**, *63*, 575–597, doi:10.1146/annurev.micro.091208.073241.
23. Traoré, D.A.K.; El Ghazouani, A.; Ilango, S.; Dupuy, J.; Jacquamet, L.; Ferrer, J.-L.; Caux-Thang, C.; Duarte, V.; Latour, J.-M. Crystal Structure of the Apo-PerR-Zn Protein from *Bacillus subtilis*. *Mol. Microbiol.* **2006**, *61*, 1211–1219, doi:10.1111/j.1365-2958.2006.05313.x.
24. Chen, L.; Helmann, J.D. *Bacillus subtilis* MrgA Is a Dps(PexB) Homologue: Evidence for Metalloregulation of an Oxidative-Stress Gene. *Mol. Microbiol.* **1995**, *18*, 295–300, doi:10.1111/j.1365-2958.1995.mmi_18020295.x.
25. Chi, B.K.; Huyen, N.T.T.; Loi, V.V.; Gruhlke, M.C.H.; Schaffer, M.; Mäder, U.; Maaß, S.; Becher, D.; Bernhardt, J.; Arbach, M.; Hamilton, C. J.; Slusarenko, A. S.; Antelmann, H. The Disulfide Stress Response and Protein S-Thioallylation Caused by Allicin and Diallyl Polysulfanes in *Bacillus subtilis* as Revealed by Transcriptomics and Proteomics. *Antioxidants* (Basel) **2019**, *8*, 605, doi:10.3390/antiox8120605.
26. Chi, B.K.; Albrecht, D.; Gronau, K.; Becher, D.; Hecker, M.; Antelmann, H. The Redox-Sensing Regulator YodB Senses Quinones and Diamide via a Thiol-Disulfide Switch in *Bacillus subtilis*. *PROTEOMICS* **2010**, *10*, 3155–3164, doi:10.1002/pmic.201000230.
27. Leelakriangsak, M.; Huyen, N.T.T.; Töwe, S.; Van Duy, N.; Becher, D.; Hecker, M.; Antelmann, H.; Zuber, P. Regulation of Quinone Detoxification by the Thiol Stress Sensing DUF24/MarR-like Repressor, YodB in *Bacillus subtilis*. *Mol. Microbiol.* **2008**, *67*, 1108–1124, doi:10.1111/j.1365-2958.2008.06110.x.
28. Palm, G.J.; Khanh Chi, B.; Waack, P.; Gronau, K.; Becher, D.; Albrecht, D.; Hinrichs, W.; Read, R.J.; Antelmann, H. Structural Insights into the Redox-Switch Mechanism of the MarR/DUF24-Type Regulator HypR. *Nucleic Acids Res.* **2012**, *40*, 4178–4192, doi:10.1093/nar/gkr1316.
29. Nakano, S.; Erwin, K.N.; Ralle, M.; Zuber, P. Redox-Sensitive Transcriptional Control by a Thiol/Disulphide Switch in the Global Regulator, Spx. *Mol. Microbiol.* **2005**, *55*, 498–510, doi:10.1111/j.1365-2958.2004.04395.x.
30. Gaballa, A.; Antelmann, H.; Hamilton, C.J.; Helmann, J.D. Regulation of *Bacillus subtilis* Bacillothiol Biosynthesis Operons by Spx. *Microbiology,* **2013**, *159*, 2025–2035.
31. Zuber, P. Spx-RNA Polymerase Interaction and Global Transcriptional Control during Oxidative Stress. *J. Bacteriol.* **2004**, *186*, 1911, doi:10.1128/JB.186.7.1911-1918.2004.
32. Nakano, S.; Nakano, M.M.; Zhang, Y.; Leelakriangsak, M.; Zuber, P. A Regulatory Protein That Interferes with Activator-Stimulated Transcription in Bacteria. *Proc Natl Acad Sci USA* **2003**, *100*, 4233, doi:10.1073/pnas.0637648100.
33. Huyen, N.T.T.; Eiamphungporn, W.; Mäder, U.; Liebeke, M.; Lalk, M.; Hecker, M.; Helmann, J.D.; Antelmann, H. Genome-Wide Responses to Carbonyl Electrophiles in *Bacillus subtilis*: Control of the Thiol-Dependent Formaldehyde Dehydrogenase AdhA and Cysteine Proteinase YraA by the MerR-Family Regulator YraB (AdhR). *Mol. Microbiol.* **2009**, *71*, 876–894, doi:10.1111/j.1365-2958.2008.06568.x.
34. Yasueda, H.; Kawahara, Y.; Sugimoto, S. Bacillus subtilis YckG and YckF Encode Two Key Enzymes of the Ribulose Monophosphate Pathway Used by Methylotrophs, and YckH Is Required for their Expression. *J. Bacteriol.* **1999**, *181*, 7154, doi:10.1128/JB.181.23.7154-7160.1999.
35. Yurimoto, H.; Hirai, R.; Matsuno, N.; Yasueda, H.; Kato, N.; Sakai, Y. HxlR, a Member of the DUF24 Protein Family, Is a DNA-Binding Protein That Acts as a Positive Regulator of the Formaldehyde-Inducible HxlAB Operon in *Bacillus subtilis*. *Mol. Microbiol.* **2005**, *57*, 511–519, doi:10.1111/j.1365-2958.2005.04702.x.
36. Au, N.; Kuester-Schoeck, E.; Mandava, V.; Bothwell, L.E.; Canny, S.P.; Chachu, K.; Colavito, S.A.; Fuller, S.N.; Groban, E.S.; Hensley, L.A.; O'Brien, T.; Shah, A.; Tierney, J. T.; Tomm, L. L.; O'Gara, T. M.; Goranov, A. I.; Grossman, A. D.; Lovett, C. M. Genetic Composition of the *Bacillus subtilis* SOS System. *J. Bacteriol.* **2005**, *187*, 7655, doi:10.1128/JB.187.22.7655-7666.2005.
37. Groban, E.S.; Johnson, M.B.; Banky, P.; Burnett, P.-G.G.; Calderon, G.L.; Dwyer, E.C.; Fuller, S.N.; Gebre, B.; King, L.M.; Sheren, I.N.; Von Mutius, L. D.; O'Gara, T. M.; Lovett, C. M. Binding of the *Bacillus subtilis* LexA Protein to the SOS Operator. *Nucleic Acids Res.* **2005**, *33*, 6287–6295, doi:10.1093/nar/gki939.
38. Chi, B.K.; Kobayashi, K.; Albrecht, D.; Hecker, M.; Antelmann, H. The Paralogous MarR/DUF24-Family Repressors YodB and CatR Control Expression of the Catechol Dioxygenase CatE in *Bacillus subtilis*. *J. Bacteriol.* **2010**, *192*, 4571, doi:10.1128/JB.00409-10.
39. Choi, H.-J.; Kim, S.-J.; Mukhopadhyay, P.; Cho, S.; Woo, J.-R.; Storz, G.; Ryu, S.-E. Structural Basis of the Redox Switch in the OxyR Transcription Factor. *Cell* **2001**, *105*, 103–113, doi:10.1016/S0092-8674(01)00300-2.
40. Zheng, M.; Åslund, F.; Storz, G. Activation of the OxyR Transcription Factor by Reversible Disulfide Bond Formation. *Science* **1998**, *279*, 1718, doi:10.1126/science.279.5357.1718.

41. Imlay, J.A. The Molecular Mechanisms and Physiological Consequences of Oxidative Stress: Lessons from a Model Bacterium. *Nat. Rev. Microbiol.* **2013**, *11*, 443.
42. Müller, A.; Eller, J.; Albrecht, F.; Prochnow, P.; Kuhlmann, K.; Bandow, J.E.; Slusarenko, A.J.; Leichert, L.I.O. Allicin Induces Thiol Stress in Bacteria through S-Allylmercapto Modification of Protein Cysteines. *J. Biol. Chem.* **2016**, *291*, 11477–11490, doi:10.1074/jbc.M115.702308.
43. Arsène, F.; Tomoyasu, T.; Bukau, B. The Heat Shock Response of *Escherichia coli*. *Inter. J. Food Microbiol.* **2000**, *55*, 3–9, doi:10.1016/S0168-1605(00)00206-3.
